# The *AalNix3&4* isoform is required and sufficient to convert *Aedes albopictus* females into males

**DOI:** 10.1371/journal.pgen.1010280

**Published:** 2022-06-23

**Authors:** Yijie Zhao, Binbin Jin, Peiwen Liu, Xiaolin Xiao, Lijun Cai, Zhensheng Xie, Ling Kong, Tong Liu, Wenqiang Yang, Yang Wu, Jinbao Gu, Zhijian Tu, Anthony A. James, Xiao-Guang Chen

**Affiliations:** 1 Department of Pathogen Biology, Institute of Tropical Medicine, School of Public Health, Southern Medical University, Guangzhou, China; 2 Department of Biochemistry and the Fralin Life Science Institute, Virginia Tech, Blacksburg, Virginia, United States of America; 3 Department of Microbiology & Molecular Genetics, University of California, Irvine California United States of America; 4 Department of Molecular Biology & Biochemistry, University of California, Irvine California United States of America; University of Michigan, UNITED STATES

## Abstract

*Aedes albopictus* is one of the most invasive insect species in the world and an effective vector for many important arboviruses. We reported previously that *Ae*. *albopictus Nix* (*AalNix*) is the male-determining factor of this species. However, whether *AalNix* alone is sufficient to initiate male development is unknown. Transgenic lines that express each of the three *AalNix* isoforms from the native promoter were obtained using *piggyBac* transformation. We verified the stable expression of *AalNix* isoforms in the transgenic lines and confirm that one isoform, *AalNix3&4*, is sufficient to convert females into fertile males (pseudo-males) that are indistinguishable from wild-type males. We also established a stable sex-converted female mosquito strain, *AalNix3&4*-♂4-pseudo-male. The pseudo-male mosquitoes can fly and mate normally with wild-type female, although their mating competitiveness is lower than wild-type. This work further clarifies the role of *AalNix* in the sex determination pathway and will facilitate the development of *Ae*. *albopictus* control strategies that rely on male-only releases such as SIT and sex-ratio distortion.

## Author summary

*Nix* serves as a conserved male-determining factor in the two most important mosquito arboviral vectors, *Ae*. *aegypti* and *Ae*. *albopictus*. *AaeNix* alone can convert *Ae*. *aegypti* females into fertile but flightless males. *AalNix* has four alternative splice isoforms whereas *AaeNix* has one. Little was known previously about which AalNix isoform(s) serve as the primary signal for sex determination. We cloned the promoter region of *AalNix* gene and constructed *piggybac-*based *AalNix* overexpression constructs with different isoform variants. Following transformation and recovery of transgenic lines, we observed that expression of the *AalNix3&4* isoform could shift the alternative splicing of the sex determination genes, *doublesex* and *fruitless*, from female to male isoforms, and phenotypically masculinize females or completely convert females into males. Importantly, the sex-converted pseudo-males are fertile and capable of flight. Thus, *AalNix* is the primary signal for male sex determination in *Aedes albopictus* and provides a basis for sex segregation and further Cas9-mediated gene-drive population suppression.

## Introduction

*Aedes albopictus* is one of the most invasive and aggressive insect species in the world [1]. It is a competent vector for several globally-important arboviruses, including dengue (DENV), Zika (ZIKV), yellow fever and chikungunya (CHIKV) viruses [2–5]. Female mosquitoes require blood meals (hematophagy) from terrestrial vertebrates for both reproduction and nutrition. The process of blood feeding allows pathogens carried by the mosquitoes to be transmitted to vertebrate hosts and this poses a serious threat to human health [6].

In some Dipteran insects, including mosquitoes and non-Drosophilid flies, the presence of products encoded by a dominant male determining factor (M-factor) is the primary initiator of sex determination. Females in these species lack this factor and are designated m/m and males are heterogametic, M/m. The *Nix* gene was first identified as the M-factor in the yellow Fever mosquito, *Aedes aegypti*, and the *AaeNix* ortholog is linked to the 1^st^ chromosome and is both required and sufficient to initiate male development [7]. Disruption of the *Ae*. *albopictus* ortholog, *AalNix*, can result in the feminization of males [8]. *Guy1* and *Yob*/*gYG2* are the candidate M-factors in the malaria vector mosquitoes, *Anopheles stephensi* and *An*. *gambiae*, respectively [9–11]. The *Mdmd* gene is the M-factor of the house fly, *Musca domestica*, and is indispensable for normal male development [12]. *MoY* is necessary and sufficient for male development and the M-factor of the fruit fly, *Ceratitis capitata* [13].

A cascade of genes in the sex-determination pathway relay the information from the primary signal to achieve terminal morphological and physiological differentiation [14]. In a previous study, we determined that the *AalNix* gene product is the primary signal for the *Ae*. *albopictus* sex-determination pathway [8]. However, whether *AalNix* alone can be used to initiate male determination requires further investigation. We also had shown that the male-specific *doublesex* gene isoform (*dsx*^*M*^) is the default splicing pattern in *Aedes* mosquito, which means that one or more potential *dsx* splicing enhancers link NIX and DSX [15]. The sex-specific splicing of *dsx* orthologues in *Drosophila* species is controlled by *transformer* (*tra)* [16–18]. Our working model is that accumulation of *AalNix*, a pre-mRNA splicing factor, is sufficient to regulate the alternative splicing of the unknown splicing enhancer, and cause this splicing factor to lose its ability to regulate *dsx* splicing, enabling *dsx* to recover its male-default splicing. However, no ortholog of *tra* has been found yet in mosquito families, most likely because it evolves rapidly resulting in significant sequence divergence [19,20]. The link of the M factor with the downstream *dsx* is still largely unexplored in mosquitoes.

Our previous work with *AalNix* showed that the gene has four exons (1–4) [8]. These exons are spliced alternately to produce four isoforms designated *AalNix1* (contains all four exons), *AalNix2* (exons 1, 3 and 4), *AalNix3* (exon 1, intron 1 and 14 base-pairs (bp) of the 5’-end of exon 2) and *AalNix4* (exon 1, intron 1 and 59 bp of the 5’-end of exon 2). *AalNix1* has two regions homologous to the *Ae*. *aegypti* ortholog, *AaeNix*, that encode two RNA-binding motifs (RRM). *AalNix2* encodes one RRM resulting from an exon 2 skip. *AalNix3* and *AalNix4* share the same stop codon due to retention of a short intron. Using mosquito transgenesis and expression constructs for each of the known *AalNix* isoforms, we show that *AalNix3&4* alone is required and sufficient to convert females into fertile males with phenotypes similar to wild-type males. We also mated pseudo-males derived from genotypically m/m females carrying only the *AalNix3&4* transgene to wild-type female mosquitoes and obtained stable lines. These studies support the further development of *AalNix* transgenes, as the male-determining factor of *Ae*. *albopictus*, for applications to sterile insect technologies (SIT) for mosquito control and provide the basis for the next step in the implementation of Cas9/guide RNA-based gene-drive systems in *Ae*. *albopictus*. Theoretically, gene-drive systems should result in the offspring of drive-system males and wild-type female mosquitoes being 50% gene-drive males, and the other 50% intersex or pseudo-males due to the introduced *AalNix* gene. The intersex do not produce offspring and pseudo-males, as good as the ones of line *AalNix3&4*-♂4 do produce some offspring including new pseudo-males, while wild-type males inheriting the gene-drive transgene have the same mating ability as wild-type males, and can mate with wild-type females to transmit the gene drive system resulting in population suppression. Our work confirms and extends a recent contribution by Lutrat et al [21].

## Results

### Generation of transgenic mosquitoes for each *AalNix* isoform

**Fig 1:**
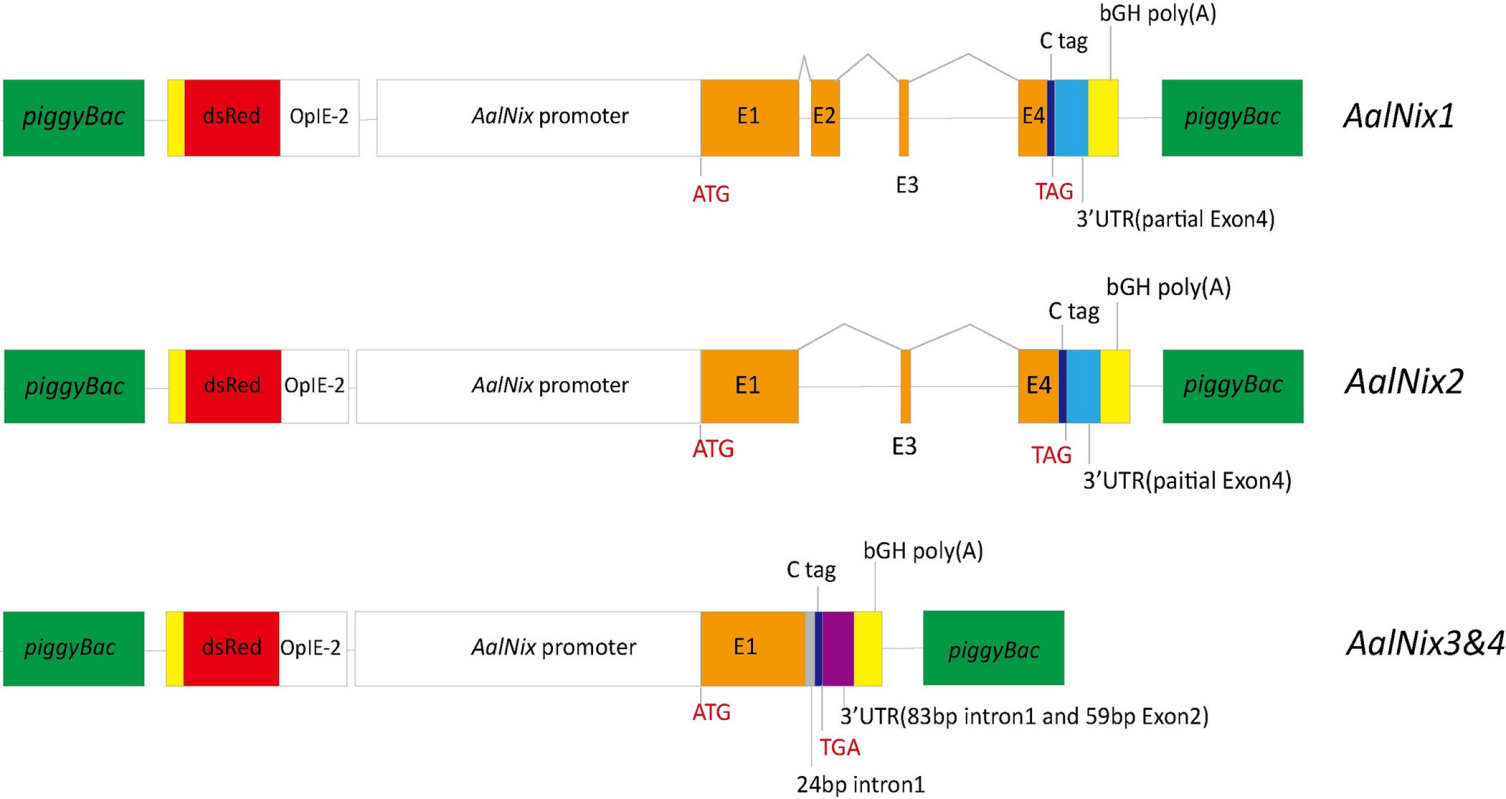
Donor plasmids used to generate transgenic *Ae*. *albopictus* expressing *AalNix* isoforms. All constructs shown in the figure are flanked by the *piggyBac* arms to facilitate transposon-mediated integration into the *Ae*. *albopictus* genome. The DsRed fluorescent marker gene under the control of the OpIE-2 promoter serves as a dominant marker gene for transformation. *AalNix* isoform variants were designed to be expressed by the *AalNix* promoter. The C-tag refers to the Strep tag II placed at the carboxyl terminus of the AalNIX protein.

**Fig 2:**
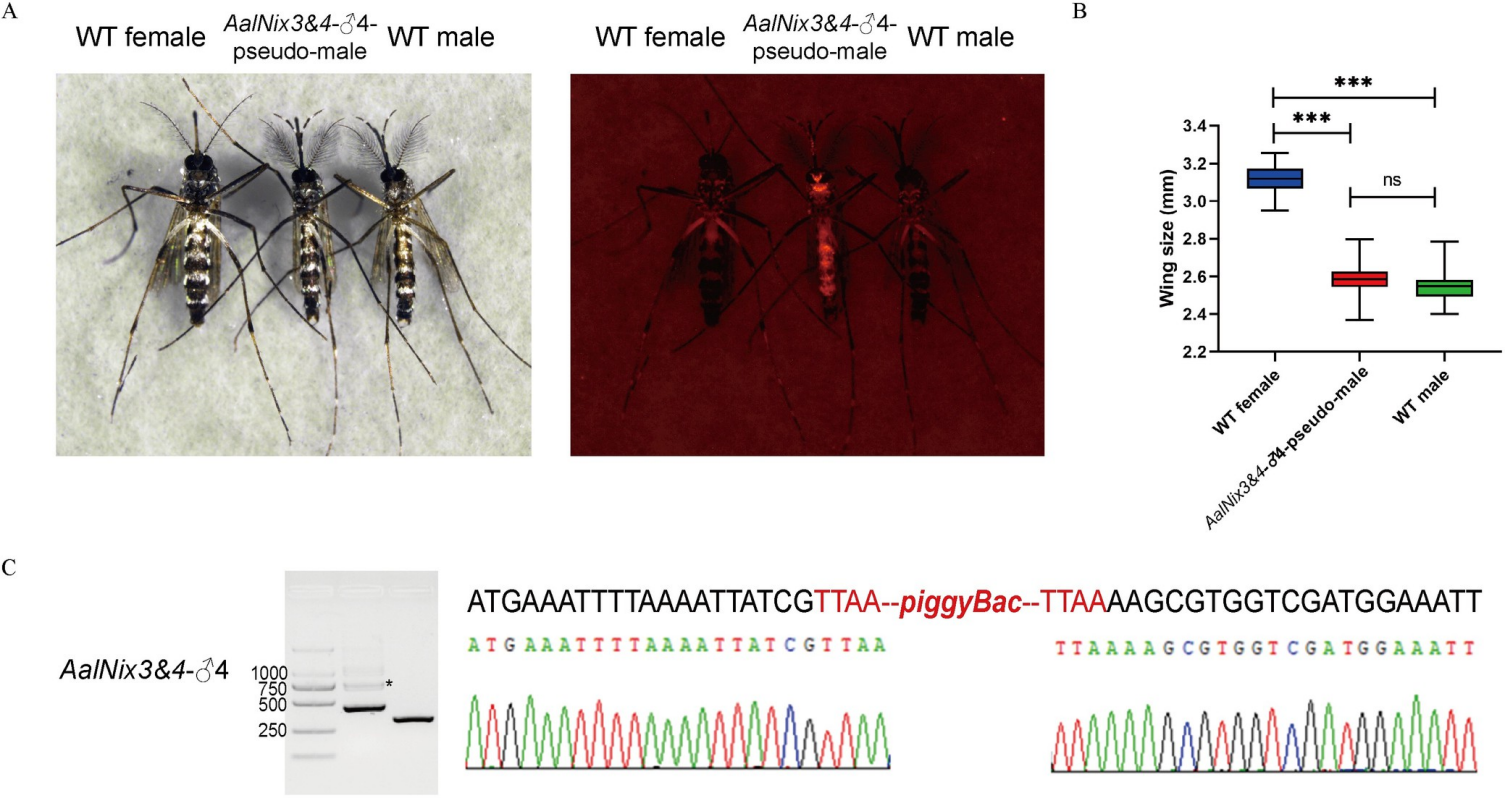
The *AalNix3&*4 transgene alone is sufficient to convert genetic females into fertile males. (A) The left panel is the bright-field image of the three genotypes of WT female, *AalNix3&4-♂4*-pseudo-male and WT male. The right panel is the florescent image of the same individuals. (B) Average length of the left and right wings of individuals of the three groups (WT female, *AalNix3&4-♂4*-pseudo-male and WT male). Thirty individuals within each group were measured from the same cohort. The box plot, starting from bottom, shows minimum values, first quartile, median, third quartile, and maximum values using horizontal solid lines, with the mean indicated by a horizontal line. (C) Analysis of the insertion site of transgenic *AalNix3&4*-♂4 strain. Agarose gel electrophoresis of PCR products showing the results of amplifying the flanking regions. Sequence analysis showed that and the insertion site is in intergenic region. The signal marked by the asterisk (*) results from incomplete digestion.

We made individual transgenes that linked the endogenous *AalNix* promoter to three distinct abbreviated forms of the coding region to see which of the four exons play roles in establishing male sex determination (Figs 1; S5). The transgenes were constructed to contain exons 1, 2, 3 and part of 4 (*AalNix*1), exons 1, 3 and part of 4 (*AalNix*2) or exon 1, intron1 and 59 bp of the 5’-end of exon 2 (*AalNix3&*4), and were inserted into a *piggyBac* transformation vector carrying a dominant fluorescent protein marker gene (OpIE2-DsRed). Following microinjection of embryos, matings of surviving injected adults (G_0_) to wild-type mosquitoes and screening of G_1_ progeny for the marker gene, three males and females each were recovered for *AalNix1* (Table 1). The line derived from one of the males, *AalNix1*-♂2, was characterized further by inverse gene amplification procedures (iPCR) and shown to be inserted into an intergenic region at AaloF1:JXUM01S001974: 263598–263603 (S1A Fig). Three transgenic males and two females were recovered in the *AalNix2* G_1_ progeny, and *AalNix*2-♂3, inserted intergenically at Aalbo_primary.1 scaffold_701: 209295–209300, was established for further analyses (S1B Fig). The G_1_ progeny arising from the *AalNix3&4* transgenesis protocols included a number of mosquitoes with an intersex phenotype arising most likely from apparent conversion of females to males and providing preliminary evidence for the activity of this isoform combination in the sex-determination pathway (Table 1). We selected 21 phenotypically-normal males, six phenotypically-normal females expressing DsRed and four intersex individuals, and two lines, *AalNix3&4*-♂4 (Aalbo_primary.1 scaffold_64: 2942049–2942054) and *AalNix3&4*-♂15 (Aalbo_primary.1 scaffold_1: 15815977–15815982), were established for further analyses (Figs 2; S1C). Both of these insertions were intergenic based on the genome annotation and did not disrupt any coding sequence, and the insertion site of *AalNix3&4-♂4* is shown in (Figs 2C; S1). Nonetheless, we do not rule out the possibility that inverse PCR cannot detect all transgene insertions in a transgenic line.

**Table 1:** Embryo microinjection results and screening of G_1_ progeny for *Nix* transformants.

Donor plasmid	# embryos injected	#G_0_ survivors (%)	# pools	# of G_1_ screened	Pools with DsRed G_1_ progeny (#)
*AalNix*1	1,150	446 (38.78%)	3	19,404	P3: (3♂; 3♀)
*AalNix*2	895	148 (16.54%)	2	9,146	P1: (3♂; 2♀)
*AalNix3&4*	1,073	150 (13.98%)	2	10,675	P2: (21♂; 6♀; 4 intersex)

### *AalNix3&4* is sufficient alone to convert females into males

We designed two pairs of oligonucleotide primers and used gene amplification analyses (PCR) to explore the inheritance profiles of the transgenic *AalNix* isoforms (S1 Table; S2A Fig). The first primer pair Nix_E1F/strep tag-R spans exon 1 and an inserted Strep II tag, and was designed to amplify the transgene insertion. The second primer pair, Nix_i2-F/Nix_i2-R, located in intron 2, are designed to distinguish endogenous *AalNix* from transgenic *Nix* (S2B Fig). Amplicons corresponding to the inserted *AalNix1* and *AalNix2* transgenes were detected in *AalNix1*-♂2and *AalNix2*-♂3, respectively, but no transgenic individuals exhibited sex conversion or intersex phenotypes (S2 and S3 Tables).

In contrast, all genetic female mosquitoes (m/m) of the *AalNix3&4*-♂4 line (confirmed by gene-specific [native vs transgenic] PCR) carrying the transgene (*Nix* presence indicated by DsRed) display only sex-converted male or intersex phenotypes (Fig 2; S4 Table). Expression of *AalNix3&4* at a site distant from the M locus results in an intersex phenotype characterized by incomplete and/or deformed male-specific, external reproductive organ-like structures in the terminal abdominal segments (S3 Fig). Interestingly, dissections of the intersex individuals revealed the presence of male-specific reproductive organs, testis and accessory glands, with abnormal morphology (S3 Fig).

The pseudo-males (m/m; *AalNix*, DsRed^+^) display complete and normal-appearing male-specific external and internal reproductive organs including gonocoxites, gonostyli, testes and accessory glands. In addition, they display the plumose antennae characteristic of wild-type males (Figs 2; S3). We selected randomly a G_2_ *AalNix3&4*-♂4 pseudo-male, mated it with wild-type females and established a stable line, *AalNix3&*4-♂4-pseudo-male, that has been propagated for >17 generations and remains stable. Remarkably, there is a complete absence of an M-locus in this cross, which was thought previously essential for the sexual development of male mosquitoes [8]. The wing-length measurements of the pseudo-male offspring of this line are comparable in size to wild-type males, supporting the conclusion that the converted males are indistinguishable from their wild-type counterparts unless viewed with fluorescence microscopy, where they display the DsRed phenotype (Fig 2B; S5 Table). All of the pseudo-males recovered in subsequent generations (G_3_-G_10_) are DsRed^+^, while the females are not, and the sex-ratio is ~1:1 (*p* = 0.865) (S6 Table). These data confirm that the *AalNix3&*4 isoform is sufficient alone to convert genotypic females (m/m) to fully phenotypic males.

The *AalNix3&4*-♂15 line yielded four phenotypes during its establishment, normal females, intersexes, converted and the wild-type males (S7 Table). The differences in the characteristics of this line when compared to *AalNix3&4*-♂4 could result from insertion site effects from it being integrated at a different location in the genome. Also, there were multiple *piggyBac* transgene insertions in these lines, and some of these insertions (at least one) were not masculinizing and produced transgenic females. Furthermore, genotypically-female (m/m), transgenic (Nix, DsRed^+^) converted males were rare (~0.5%, 21 of 4102) and the strain went extinct at 10 generations.

### Transgene-expressed *AalNix* alters *doublesex (dsx*) and *fruitless* (*fru*) splicing

Endogenous *AalNix* expression is first detected in embryos between 4h and 8h after oviposition and thereafter throughout subsequent life stages. Importantly, transcription of *AalNix* is limited to male mosquitoes and regulates the splicing of the zygotic *Aaldsx*^*M*^ isoform [8]. We investigated the impact of exogenously-supplied *AalNix* on *dsx* and *fru* to determine its impact in regulating the sex determination pathway in the transgenic mosquitoes.

**Fig 3:**
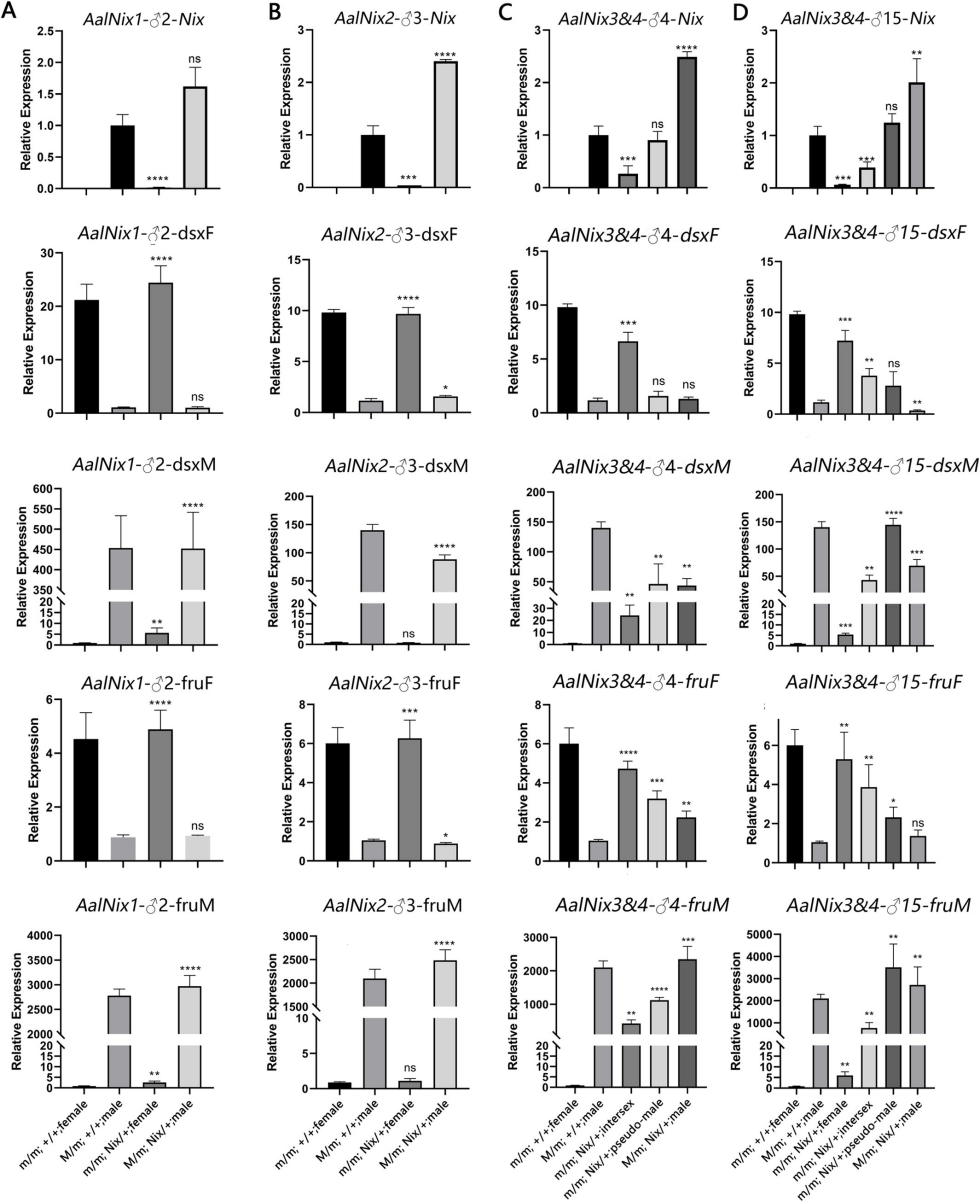
Quantification (qRT-PCR analysis) of relative mRNA levels of *AalNix* and downstream genes in wild-type males and females, and each phenotypic of transgenic mosquito in each strain. *AalNix*, *Aalbdsx* and *Aalfru* of *AalNix1*-*♂*2(A), *AalNix2*-♂3 (B), *AalNix3&4*-♂4 (C) and *AalNix3&4*-♂15 (D). The results were normalized to the *Ae*. *albopictus Rps7* (*AalRpS7*) gene and are shown as the mean ± SD of three technical replicates.*P<0.05;**P<0.01; ***P<0.005; ****P<0.0001.

*AalNix* expression in *AalNix1*-♂2 and *AalNix2*-♂3 transgenic males was 1.62-fold and 2.40-fold higher than wild-type males, respectively, while as expected, *AalNix* transcripts are not found in transgenic females (Fig 3). As a downstream gene of sex determination pathway, the female and male isoforms of *dsx* and *fru* do not change in the *AalNix* transgenic individuals, compared to wild-type individuals (Fig 3).

The expression levels of *AalNix* gene transcripts in *AalNix3&4*-♂4 (carrying the extra copy of the expressed first exon and 24 bp of intron 1) intersex individuals, pseudo-males and male were 0.27-, 0.9- and 2.48-fold greater, respectively, than wild-type males (Fig 3A–3D). The female-specific *dsx*^*F*^ splice variant was the main isoform detected in wild-type females, and the male-specific splicing variant, *dsx*^*M*^ was predominant in males. Interestingly, *dsx*^*F*^ transcripts are abundant in intersex mosquitoes, which are phenotypically more similar to wild-type females. Furthermore, the transcript accumulation of *dsx*^*F*^ in pseudo-males is 0.16-fold lower than wild-type females and is closer to that of wild-type males. The transcript accumulation levels of the *dsx*^*M*^ isoform in intersexes is 17% that of wild-type male mosquitoes and 24-fold higher than of wild-type females. However, *dsx*^*M*^ accumulates to similar levels in pseudo-males and genotypic males (M/m) carrying the *AalNix3&4* transgene and these are lower than in wild-type males. The expression trends of *fru*^*F*^ and *fru*^*M*^ are similar to *dsx*^*F*^ and *dsx*^*M*^ (Fig 3A–3D).

### Mating competitiveness of transgenic male mosquitoes

Competition assays in cages involved exposing ten wild-type females to mixed groups of pseudo-males, transgenic males expressing both native *AalNix* and exogenous *AalNix3&4* and wild-type males. Following exposure to a 1:1 ratio of five pseudo-males to five wild-type males, the percentage of females that produced broods that contained transgenic progeny was 15% (9 of 51) (Table 2) and the percentage of transgenic progeny was 9.08% (251 of 2,512) (S8 Table). Although the visible morphological phenotypes of the transgenic males are not distinguishable from those of wild-type males (Fig 2), they contribute significantly fewer progeny than the wild-types to the next generation (*p* < 0.001; Table 3).

**Table 2:** Proportion of females of the *AalNix3&4*-♂4 line that produced broods with transgenic progeny.

	Brood with transgenic progeny	Brood with no transgenic progeny	Observed proportion(%)
Exp1^1^ (m/m; Nix/+) pseudo-male: (M/m; +/+) WT♂ = 1:1	9	51	15.0%
Exp2^2^ (M/m; Nix/+) male: (M/m; +/+)WT♂ = 1:1	35	25	58.3%

1. 5 pseudo-male mosquitoes mixed with 5 WT male, and then mated with 10 unmated WT female mosquitoes

2. 5 transgenic male mosquitoes mixed with 5 WT male, and then mated with 10 unmated WT female mosquitoes

**Table 3:** Proportion of transgenic progeny in *AalNix3&4*-♂4 line competition experiments.

Replicate	DsRed^+^	DsRed^-^	Percent
1	25	436	5.42
2	34	398	7.87
3	31	403	7.14
4	77	543	12.42
5	59	424	12.22
6	25	308	7.51
Total	251	2512	9.08

Five *AalNix3&4*- ♂4 pseudo-male (m/m, ♂) compete with five wild-type males (M/m).

*(*p* < 0.001)

A competition experiment in cages (six replicates) involving exposing ten wild-type females to a 1:1 ratio of wild-type: heterozygous transgenic males (M/m; Nix/+) resulted in 58.3% (35 of 25) (Table 2) of the females producing broods with transgenic progeny of which 31.89% (603 of 1,288) (*p* < 0.001) were transgenic (Table 4; S9 Table). This data support the further development of these or similar transgenic strains for application to future population replacement or population suppression genetic control strategies.

**Table 4:** Proportion of transgenic progeny.

Replicate	DsRed^+^	DsRed^-^	Percent (%)
1	86	247	25.83
2	94	271	25.75
3	144	180	44.44
4	93	225	29.25
5	95	200	32.20
6	91	165	35.55
Total	603	1288	31.89*

Five *AalNix3&4*-♂4 male(M/m, ♂) compete with five wild type males.

*(*p* < 0.001)

## Discussion

The results of these experiments demonstrate that the expression of exogenously-derived *AalNix* gene products can convert genotypically (m/m) *Ae*. *albopictus* females into males. Transgenic females carrying and expressing the first *AalNix* exon can develop into functional adult males capable of mating with wild-type females to produce normal offspring. These data are consistent with a recent report and confirm that the *Nix* gene alone is sufficient to initiate male development in *Ae*. *albopictus*, and lays the foundation for the next step to link the *Nix* gene with a CRISPR/Cas9 gene-drive system for control of vector-borne diseases.

*Aedes albopictus* and *Ae*. *aegypti* have a sex-determining locus, M, on their 1^st^ chromosomes. This chromosome behaves generally in all other aspects as an autosome. There are two annotated protein-encoding genes in the *Ae*. *aegypti* M-locus, including *Nix*, and a *myo-sex* gene that has a role in the flight of adult males [22]. However, only one gene orthologous with *AaeNix* has been found in the M-locus of *Ae*. *albopictus* and it is designated *AalNix* [23]. Unlike *AaeNix*, which has only a single isoform, *AalNix* has four isoforms (*AalNix1-4*). The coding sequence of *AalNix3* and *AalNix4* are nearly identical and contain only the first RRM motif found in *AaeNix* [8]. Intron 1 and its 5’-end donor site location is conserved among *AaeNix* and *AalNix* (S4 Fig). It is worth noting that *AalNix3* and *AalNix4* differ from other isoforms in that their CDS includes 24 bases of the *AalNix* gene intron1. As an RRM motif-containing protein, NIX is critical for *dsx* and *fru* male-specific transcript splicing in the sex-determination pathway. We constructed three *AalNix* isoform expression plasmids and were able to establish the transgenic strains of each isoform. Expression of exogenously-provided *AalNix1* and *AalNix2* did not result in sex conversion in the females in the corresponding transgenic lines, but we did not recover a larger number of lines. In contrast, genetic females (m/m) of *AalNix3&4* lines showed conversion to morphologically-normal wild-type (M/m) males. The different *AalNix3&4* insertion sites in two distinct transgenic lines produced genetic females (m/m) with different degrees and proportions of male conversion, most likely resulting from an insertion-site effects. In addition, it is known that using *piggyBac* is likely to produce multiple-copy insertions [24], and inverse PCR mapping in lines most-likely detected only one of the multiple insertions, not necessarily the masculinizing one, results in the differences observed between *AalNix3&*4-♂4 and *AalNix3&*4-♂15. As seen with previous work with *AalNix* gene [8], the *AalNix3&4* isoform was found in male pupae, male adults and C6/36 cells, supporting the conclusion that it plays a major role in the development of sex characteristics of *Ae*. *albopictus* males. While it cannot be ruled out that the insertion-sites affect *AalNix1* and *AalNix2* function because both contain exon 1, *AalNix3&4* has the extra 25 bp of intron 1 and contains only one RRM site. It is possible that *AalNix1* and *AalNix2* may have roles assisting *AalNix3&*4 in the male sex determination pathway. As shown in the mating competition experiment, wild-type males carrying the transgene were as competitive as those without the transgene, but the mating competitiveness of pseudo-males was reduced.

We also isolated a completely gender-converted female from the *AalNix3&*4-♂4 strain, and following mating it with wild-type females to establish a stable strain, all subsequent male progeny of outcrosses to wild-type females inherited the *AalNix3&4* transgene (DsRed^+^), while the females did not (DsRed^-^). Thus, we have inserted an artificial M-locus into the female (m/m) genome and produced fully-functional males through sex conversion.

The strain carrying the male sex-linked fluorescent markers are ideally suited for automated fluorescence sex-sorting needed for technologies such as SIT where it is important to obtain large numbers of male mosquitoes. Furthermore, since *Nix* alone completely converts females to males, combining it with a gene-drive system could produce a strain in which all resulting progeny are males following outcrossing [19,25]. We are working now to build such a gene-drive system in *Ae*. *albopictus* based on gender conversion and this could help achieve the goal of eliminating breeding female mosquitoes by producing pseudo-male mosquitoes.

## Materials and methods

### Mosquitoes

The *Ae*. *albopictus* Foshan strain was a kind gift from the Centers for Disease Control of Guangdong Province. This strain was isolated from Foshan, Guangdong, PRC and has been established in the laboratory since 1981. All mosquitoes are reared in an insect breeding room with constant temperature and humidity (27 ± 1°C, relative humidity 70%-80%) under a 14 L:10D photoperiod. The larvae were reared in plastic pots and fed ground turtle food (INCH-GOLD, Shenzhen, China). Adult mosquitoes were kept in 20 X 20 X 30 cm nylon mesh cages and allowed access to a cotton wick soaked in a 10% glucose solution and mated freely. Adult females were allowed to take blood meals from defibrinated sheep blood (Solarbio Life Sciences, Beijing, China) using a Hemotek membrane feeding system (Discovery Workshops, Accrington, UK).

### Plasmid construction

All oligonucleotide primers are listed in S1 Table. To generate the pAalNix constructs, the *piggyBac* backbone plasmid AAEL010097-Cas9 (a kind gift from Omar Akbari [Addgene plasmid # 100707; http://n2t.net/addgene:100707]) [3] was digested and ligated to a linearized *AalNix* cassette (containing the CDS of AalNix1, AalNix2, AalNix3) at *Not* I/*Asc* I sites using the In-Fusion HD Cloning Kit (Takara, Dalian, China) [26]. To construct the *piggyBac*-AalNix1 plasmid, a 3,967-bp sequence containing the 2,478-bp *AalNix* native promoter (S5 Fig) (including the 133-bp 5′-end UTR), the 999-bp Nix1 ORF (the 24-bp Strep-tag II fusion to the C-terminus of *AalNix1*), and a 239 bp 3′-end UTR region followed by the bGH poly(A) signal, was amplified from pAalNix1 [15] using the primers Aal01 and Aal02 and cloned into the linearized backbone vector described above. To construct the *piggyBac*-AalNix2 plasmid, the backbone of the AAEL010097-Cas9 Vector and two DNA fragments were then joined in the following order: 1) *AalNix* native promoter and exon1 sequence (674bp) was amplified from pAalNix1 using the primer Aal03 and Aal04; 2) the sequence of the remaining 247-bp *AalNix2* ORF region (Strep-Tag II fusion to the C-terminus of *AalNix2*) and 159 bp 3′-end UTR region followed by bGH poly(A) signal (this fragment was synthesis *in vitro* (Invitrogen, Guangzhou, China) using primers Aal05 and Aal06. These two fragments were purified and joined using the In-Fusion HD Cloning Kit (Takara), resulting in the pAalNix2 plasmid. The pAalNix3&4 plasmid was constructed using the same method for the pAalNix2 plasmid: 1) the *AalNix* native promoter and exon1 sequence were amplified from pAalNix1 using the primer Aal07 and Aal08; 2) the sequence of the remaining 25-bp AalNix4 ORF region (Strep-Tag II fusion to the C-terminus of AalNix4) and 138 bp 3′ UTR region followed by bGH poly-(A) signal sequence (Invitrogen Gene Synthesis service) using primers Aal09 and Aal10. These two fragments were purified and inserted into pAalNix3&4 plasmid using the In-Fusion HD Cloning Kit (Takara).

All amplification steps were performed using Q5 Hot Start High-Fidelity 2X Master Mix (NEB, Ipswich, MA) and the amplicons were analyzed by agarose gel electrophoresis and, then purified using the GeneJET Gel Extraction Kit (Thermo Fisher Scientific, Carlsbad, CA, USA). All pAalNix plasmid sequences were verified using Shenggong Biotech Sanger sequencing services (Shenggong Biotechnology Co. Ltd., Guangzhou, China). Plasmids with the correct sequence were used as donors for construction of transgenic mosquitoes.

### Generation of *Ae*. *albopictus* transgenic lines

The injected plasmid DNA mixture comprised a construct donor plasmid (500 ng/μL) and the phsp-Pbac helper plasmid [19,27,28] (300 ng/μL, a kind gift from Anthony A. James) mixed in injection buffer [29] and co-injected into 0-1h old embryos [30]. After microinjection, 16-20h old injected egg were heat shocked at 37°C for 1h, and then placed at 28°C, 80% humidity until hatching.

### Characterization insertion site of the transgenic line

Inverse PCR was performed to characterize the insertion site as described previously [3,22]. Genomic DNA was extracted from adult females and males from each line, using a DNeasy Blood and Tissue kit (Qiagen, Hinden, Germany) according to the supplier’s protocol. One microgram of DNA was digested for 8h at 37°C with *Sau3A*I (5’-end reaction) or *HinP1*I (3’-end reaction) restriction enzymes (NEB), and then purified using the GeneJET PCR Purification Kit (Thermo Fisher Scientific) in accordance with the manufacturer’s protocol. Approximately 200 ng DNA fragments were circularized with T4 DNA ligase (NEB) at 16°C overnight in a 200 uL reaction volume. Finally, the ligation product was purified and subjected to two rounds of PCR reaction using Q5 Hot Start High-Fidelity 2X Master Mix (NEB) with primers AE049-AE056 [3]. Primers Aal049/ Aal050 and primers Aal053/ Aal054 were used for the first round of PCR for 5’ -end reaction and 3’-end reaction respectively. Primers Aal051/ Aal052 and primers Aal055/ Aal056 were used for nested PCR for 5’-end reaction and 3’-end reaction, respectively. The nested PCR products were directly loaded on agarose gels, and were extracted using the GeneJET Gel Extraction Kit (Thermo Fisher Scientific), ligated using the CloneJET PCR Cloning Kit (Thermo Fisher Scientific), and transformed into DH5α competent cells (Takara). Positive clones were purified (EZNA Plasmid Mini Kit I, Omega) and sent for sequencing (ShengGong Biotechnology Co. Ltd., Shanghai, China).

### Identification of endogenous and transgenic *AalNix*

To ensure that all transgene cassettes were stably inherited to the next generation, male phenotype individuals with DsRed fluorescence were selected and a part of the leg cut off to extract DNA, using the Extract-N-Amp Tissue PCR Kit (Sigma-Aldrich Inc., St. Louis, MO) according to the manufacturer’s protocol. PCR was performed to characterize the native *AalNix* and the exogenous transgenic *AalNix* with the primers Nix_i2-F/ Nix_i2-R and the primers Nix_E1F/ strep tag-R, using DreamTaq Hot Start Green PCR Master Mix (2X) (Thermo Fisher Scientific, Waltham, MA, USA) according to the user manual. Males containing the endogenous *AalNix* and exogenous *AalNix* genes were mated with wild-type females to established lines.

### Wing-length measurement and statistical analysis

Wing-length values were measured for 30 individuals from wild-type females and males and *AalNix3&4*-♂4-pseudo-males. The wings of one mosquito were removed and placed under a laboratory microscope (connected to a computer and a camera) to take photographs. Image-pro Plus software was used to measure the wing length of adult mosquitoes. The wing length as determined is the distance from the axillary incision to the apical margin (excluding the wing-margin fringe hairs). The average length of a pair of wings from each mosquito was recorded as the wing length.

### RT-qPCR analysis of transgenic mosquitos

The relative expression levels of *AalNix*, *Aalbdsx* and *Aalfru* in transgenic mosquitos were quantified by real-time PCR using the SuperReal PreMix Plus kit (SYBR Green) (Tiangen Biotech Co., Ltd., Beijing, China) according to the manufacturer’s instructions. Total RNA was extracted from whole adult transgenic mosquitoes from each phenotype of each line, using TRIzol (Life Technologies, Carlsbad CA, USA) according to the manufacturer’s directions. Each sample contains nine adult mosquitos assessed in triplicate and normalized with AalRpS7 mRNA. The qPCR results were analyzed using the 2^−ΔΔCT^ method.

### Mating competition assays

To assess mating competitiveness between wild type and transgenic males (m/m♂; nix/+), five WT males and five transgenic males were placed into a 1.5-L container and allowed to adapt to the environment. A total 10 virgins females previously fed with a blood meal were put into the container to mate. After two day of full mating, the female mosquitoes in each container were placed in a single oviposition cup to lay eggs and these were allowed to hatch. When the hatched larvae reached the third-fourth instar, the were screened with fluorescence microscopy (Nikon SMZ1000) and counted. Six replicate cages were set up and assayed for each line as described.

### Statistical analysis

All data were represented as means ± standard deviation (SD) from at least three independent experiments. Analysis of differences between groups were analyzed by unpaired Student’s t-test. One-Way ANOVA of variance was used to evaluate spatiotemporal expression profiles of *AalNix*. Statistical significance was set at P < 0.05 and was determined using GraphPad Prism Version 8.02 program (GraphPad Software, CA, USA) and IBM SPSS Statistics 20.0 (SPSS Software, IL, USA). Gene expression was normalized using the deltaCT method against *AalRpS7* as a reference gene.

## Supporting information

S1 Fig Analysis of the insertion sites of *AalNix* isoform transgenic lines. (a) *AalNix* in the isoform1-♂1 strain. (b) *AalNix* in the isoform2-♂3 strain. (c) *AalNix* in the isoform4-♂15 strain. Agarose gel electrophoresis of PCR products showing the result of amplifying the flanking regions. Sequence analysis showed that transgenic line insertions are in intergenic regions. The band represented by the asterisk (*) results from incomplete digestion.
(TIF)

S2 Fig Location of primers for sex identification and endogenous and introduced Nix gene transcripts in transgenic and non-transgenic mosquitoes. (a) The primer pair Nix_i2-F/Nix_i2-R is located in intron 2 and were designed to distinguish the endogenous *AalNix* from the transgenic Nix. (b) Primer pair Nix_E1F/strep tag-R that span the exon1 and Strep II tag were designed to confirm transgene insertion.
(TIF)

S3 Fig Representative images showing masculinization and external deformities in sexually-dimorphic organs resulting from expression of exogenously-provided isoforms AalNix3&4. Deformities and masculinization in internal reproductive organs in transgenic female mosquitoes, we define this phenotype of mosquitoes as intersex. External genitalia (top panels), and internal genitalia (bottom panels).
(TIF)

S4 Fig Protein alignment of *AalNix3&4* and *AaeNix*. Red font peptide indicates *AalNix3&4* intron1. Green front indicates *AaeNix* Exon2.
(TIF)

S5 Fig Sequence of AalNix promoter. (TIF)

S1 Table List of oligonucleotide primers. (DOCX)

S2 Table Progeny screening of the *AalNix1*-♂2 transgenic line. (DOCX)

S3 Table Progeny screening of the *AalNix2*-♂3 transgenic line. (DOCX)

S4 Table Progeny screening of *AalNix3&4*-♂4 transgenic lines. (DOCX)

S5 Table Average length (millimeters) of the left and right wings of individuals of the WT female, *AalNix3&4*- ♂4 pseudo-male and WT male. (DOCX)

S6 Table Progeny screening of *AalNix3&4*-♂4 converted transgenic line. (DOCX)

S7 Table Progeny screening of *AalNix3&4*-♂15 transgenic line. (DOCX)

S8 Table DsRed fluorescent phenotypes in progeny resulting from competition assays of *AalNix3&4*-♂4 pseudo-males (m/m; Nix/+) and wild-type males (M/m). (DOCX)

S9 Table DsRed fluorescent phenotypes in progeny resulting from competition assays of wild-type males (M/m) with the *AalNix3&4*-♂4 transgene and wild-type (M/m) males. (DOCX)
