# Supplementary figures and images for: The AalNix3&4 isoform is required and sufficient to convert Aedes albopictus females into males
Source: PLoS Genet. 2022 Jun 23;18(6):e1010280. doi: 10.1371/journal.pgen.1010280 (PMC9258803; doi:10.1371/journal.pgen.1010280)

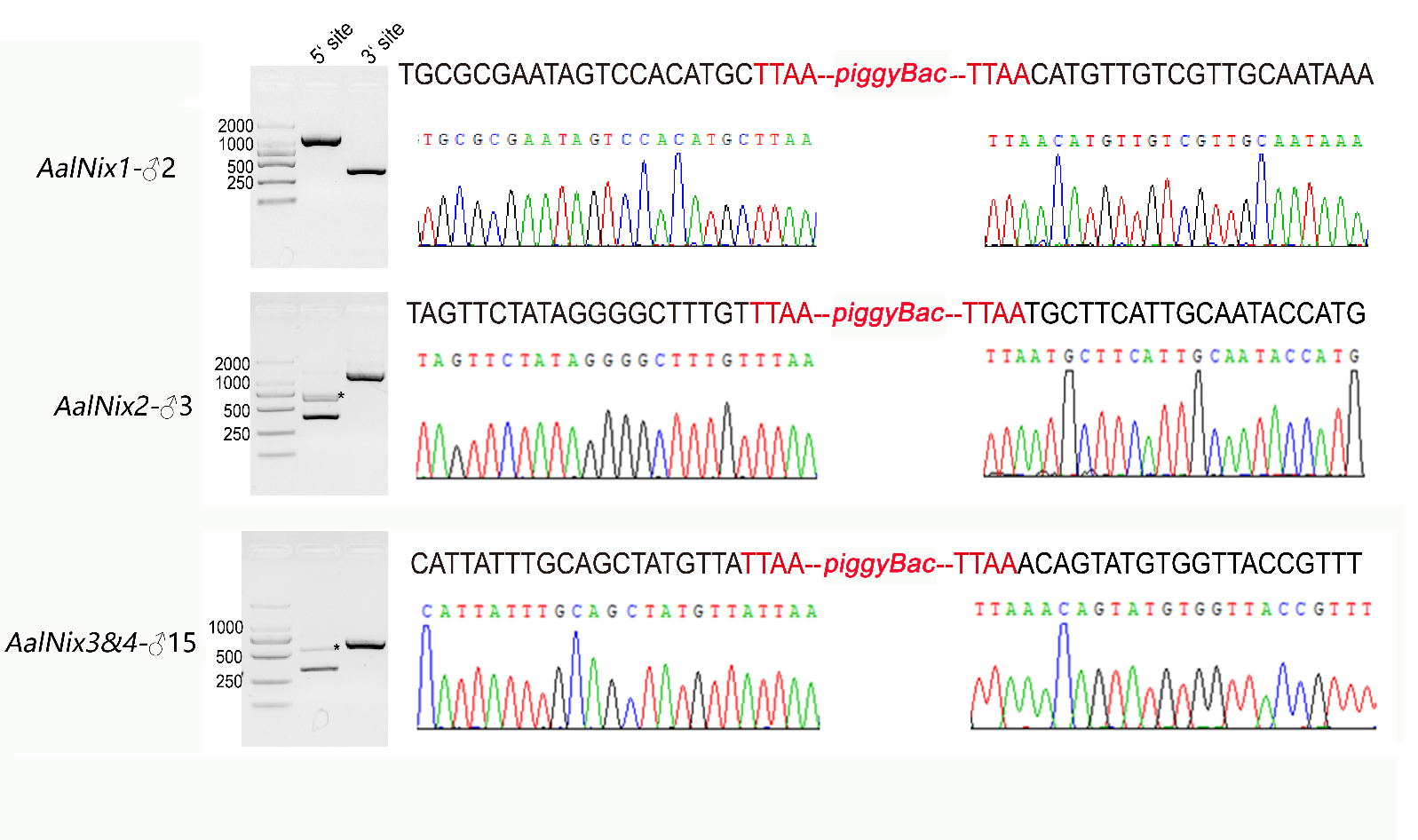

Supplement: S1 Fig — (a) AalNix in the isoform1-♂1 strain. (b) AalNix in the isoform2-♂3 strain. (c) AalNix in the isoform4-♂15 strain. Agarose gel electrophoresis of PCR products showing the result of amplifying the flanking regions. Sequence analysis showed that transgenic line insertions are in intergenic regions. The band represented by the asterisk (*) results from incomplete digestion. (TIF) [file pgen.1010280.s001.tif]

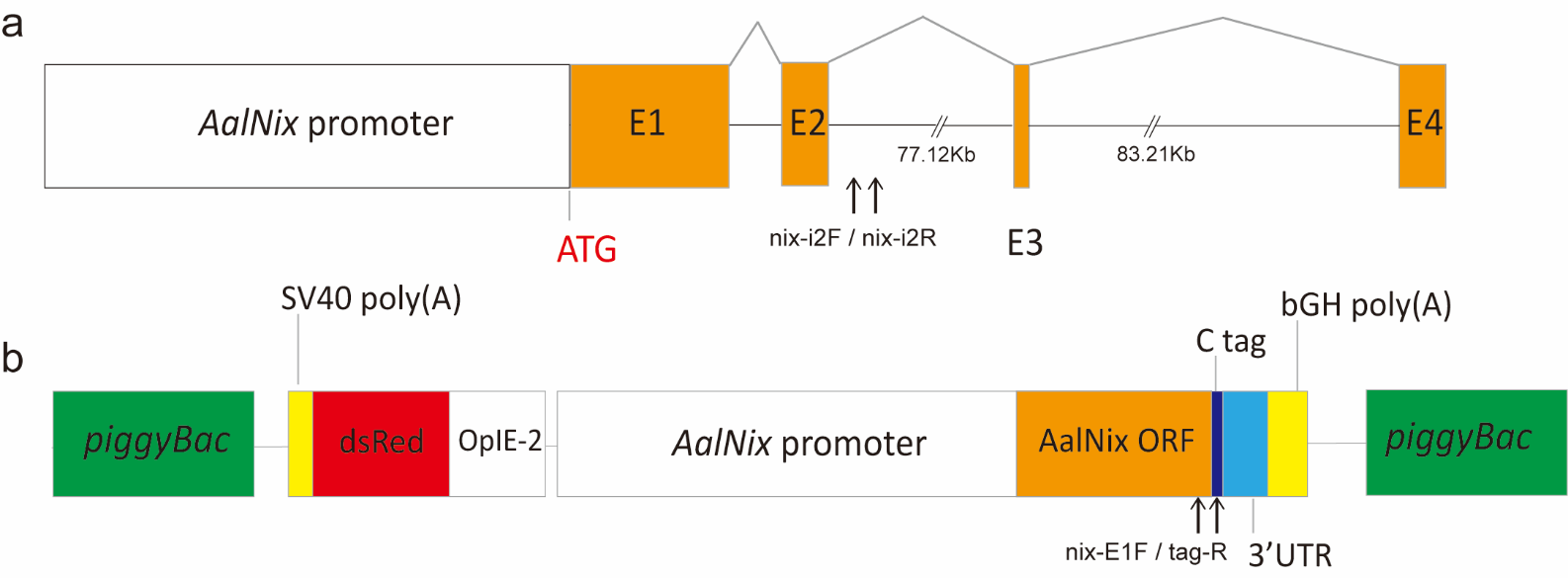

Supplement: S2 Fig — (a) The primer pair Nix_i2-F/Nix_i2-R is located in intron 2 and were designed to distinguish the endogenous AalNix from the transgenic Nix. (b) Primer pair Nix_E1F/strep tag-R that span the exon1 and Strep II tag were designed to confirm transgene insertion. (TIF) [file pgen.1010280.s002.tif]

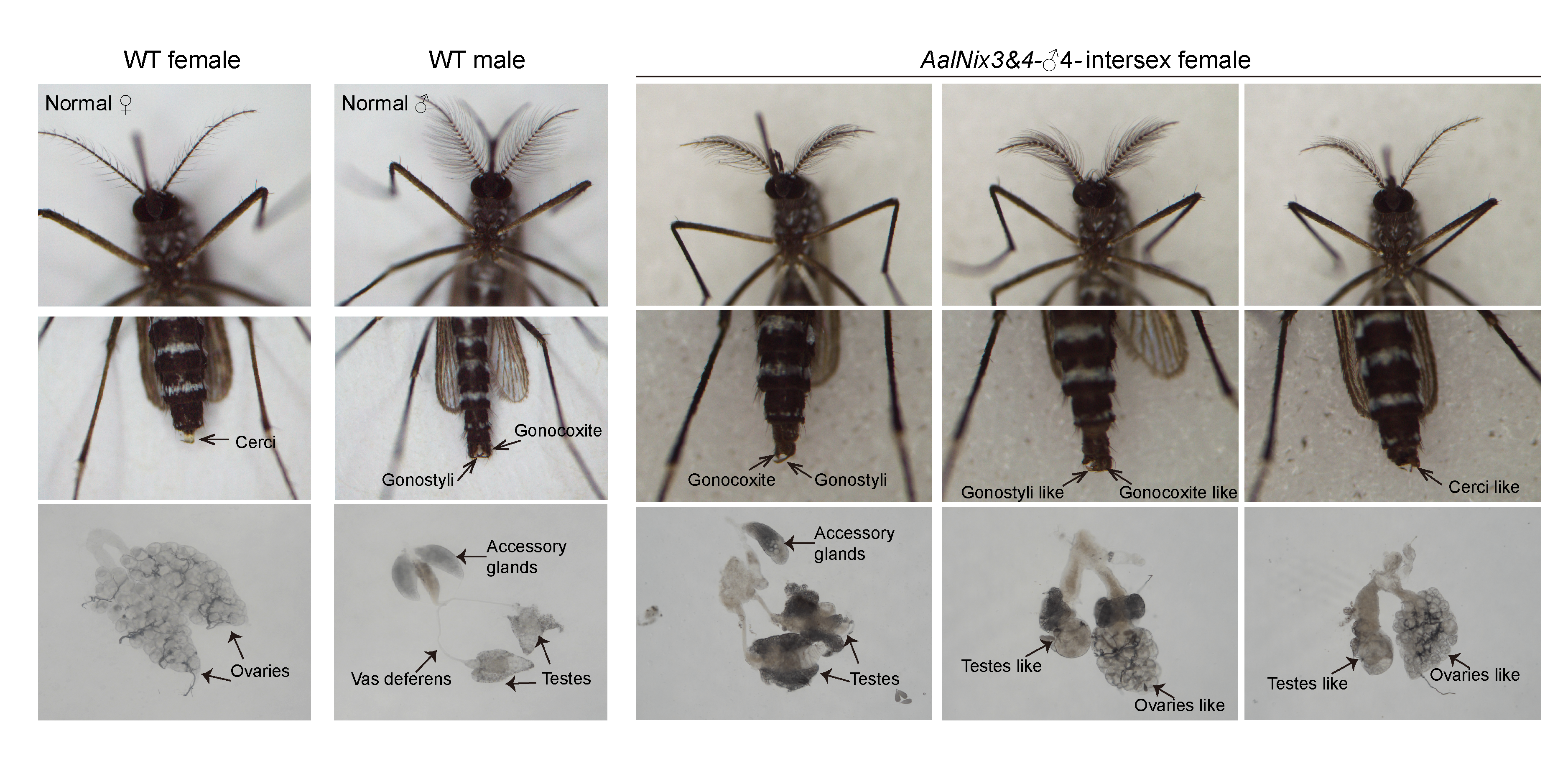

Supplement: S3 Fig — Deformities and masculinization in internal reproductive organs in transgenic female mosquitoes, we define this phenotype of mosquitoes as intersex. External genitalia (top panels), and internal genitalia (bottom panels). (TIF) [file pgen.1010280.s003.tif]

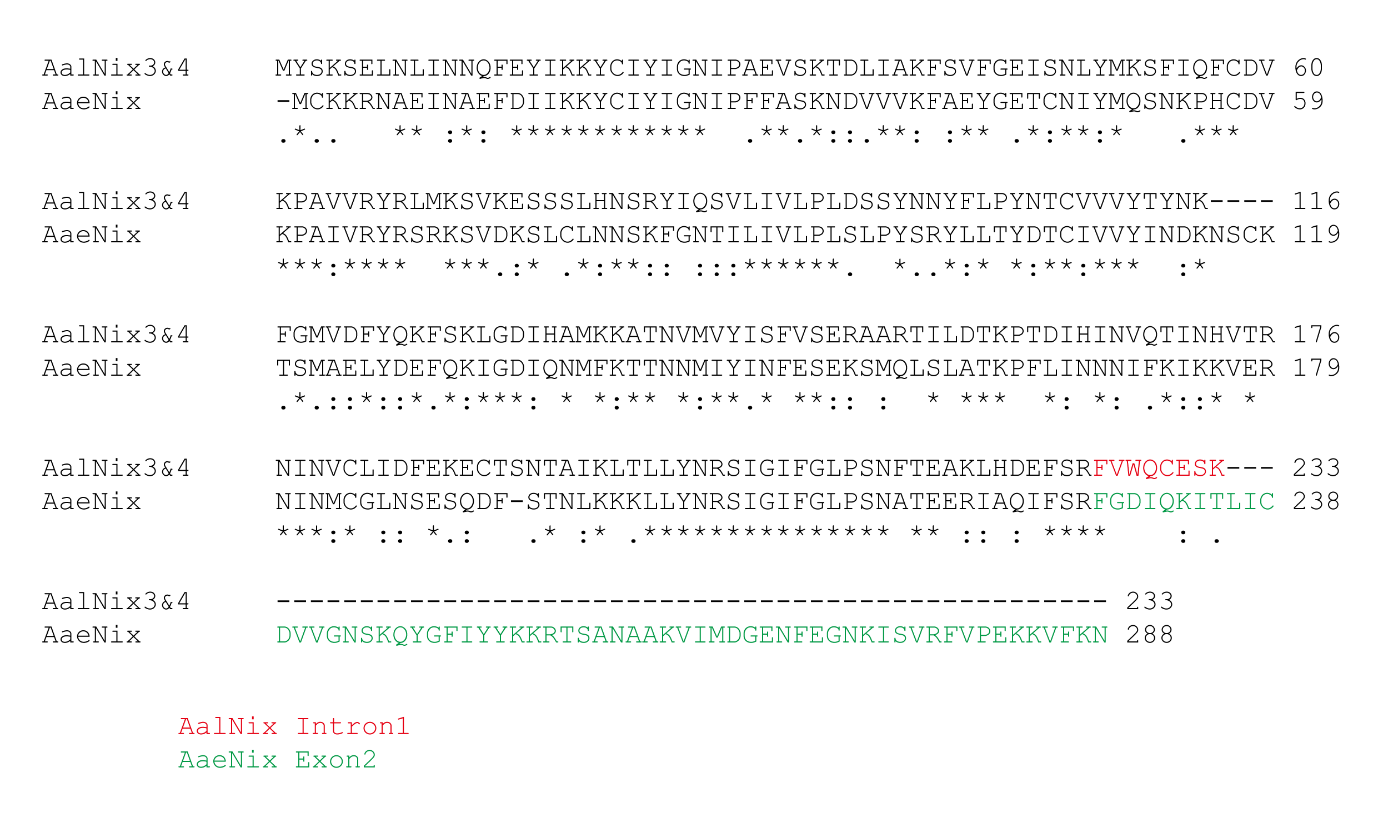

Supplement: S4 Fig — Red font peptide indicates AalNix3&4 intron1. Green front indicates AaeNix Exon2. (TIF) [file pgen.1010280.s004.tif]

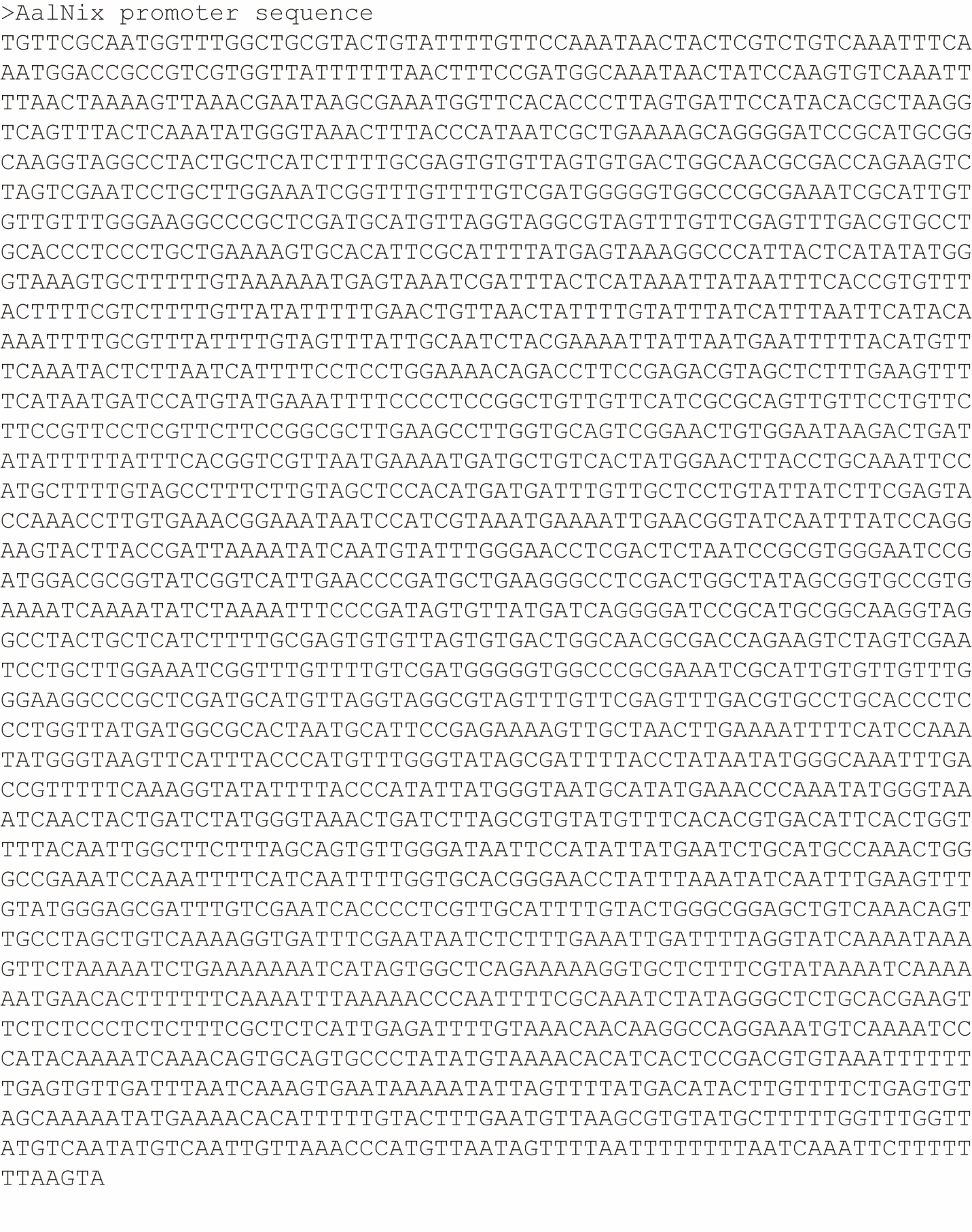

Supplement: S5 Fig — (TIF) [file pgen.1010280.s005.tif]
