# Supplementary material for: The AalNix3&4 isoform is required and sufficient to convert Aedes albopictus females into males
Source: PLoS Genet. 2022 Jun 23;18(6):e1010280. doi: 10.1371/journal.pgen.1010280 (PMC9258803; doi:10.1371/journal.pgen.1010280)
Supplement: S1 Table — (DOCX) [file pgen.1010280.s006.docx]

| **S1 Table. List of oligonucleotide primers.** | | | |
| --- | --- | --- | --- |
| **Primer** | **Sequences （5’-3’）** | **Annealing temperature and cycles** | **Primer use** |
| Aal01 | GGCCACCGAGTATGGGCGCGCCTGTTCGCAATGGTTTGGCTG | 65℃, 30 | Construct pAalNix-Nix1 plasmid |
| Aal02 | CATGTCGACGCGGCCGATATCCCATAGAGCCCACCG |  |  |
| Aal03 | GGCCACCGAGTATGGGCGCGCCTGTTCGCAATGGTTTGGCTG | 64℃, 30 | Construct pAalNix-Nix2 plasmid |
| Aal04 | CGGTCCGCAGCTTGAAAATTCATCGTGCAGTTTTG |  |  |
| Aal05 | AATTTTCAAGCTGCGGACCGTTCAAGAAATTTTG | 68℃, 30 |  |
| Aal06 | gcgggcatgtcgacgcggccgcccatagagcccaccgcatc |  |  |
| Aal07 | GGCCACCGAGTATGGGCGCGCCTGTTCGCAATGGTTTGGCTG | 64℃, 30 | Construct pAalNix-Nix3&4 plasmid |
| Aal08 | CCACACAAACCTTGAAAATTCATCGTGCAGTTTTG |  |  |
| Aal09 | aattttcaaggttTGTGTGGCAATGTGAATCC | 66℃, 30 |  |
| Aal10 | gcgggcatgtcgacgcggccgcccatagagcccaccgcatc |  |  |
| Aal049 | GACGCATGATTATCTTTTACGTGAC | 65℃，25 | 5’reaction; First round of PCR |
| Aal050 | TGACACTTACCGCATTGACA |  |  |
| Aal051 | GCGATGACGAGCTTGTTGGTG | 70℃，35 | 5’reaction; nest PCR |
| Aal052 | TCCAAGCGGCGACTGAGATG |  |  |
| Aal053 | CAACATGACTGTTTTTAAAGTACAAA | 60℃，25 | 3’reaction; First round of PCR |
| Aal054 | GTCAGAAACAACTTTGGCACATATC |  |  |
| Aa055 | CCTCGATATACAGACCGATAAAAC | 63℃，35 | 3’reaction; nest PCR |
| Aal056 | TGCATTTGCCTTTCGCCTTAT |  |  |
| Nix_i2-F | ATTCACACCATCGAAATCATCAAT | 51℃，43 | Identification of the gender transgenic strains |
| Nix_i2-R | TGCGCTCCGTGTGATACTA |  |  |
| Nix_E1F | AAGAGCTGCAAGGACCATTCT | 52℃，43 | Identification of the gender transgenic strains |
| strep tag-R | TCAAACTGCGGATGGCTCCA |  |  |
| AalrpS7 F | CTGATGCGTTCGAGGGTCAA | 55℃，43 | Reference gene for control with qPCR |
| AalrpS7 R | ACGCTCACCAATGAACACGA |  |  |
| Aalbdsx♂qF | CAGCAGCTCCTGGAGAA | 55℃，40 | Expression profile analysis for *dsx^M^* / *dsx^F^* with qPCR |
| Aalbdsx♂qR | ACAGAGGTCGTGTGCTT |  |  |
| Aalbdsx♀qF | ATCAAGTCAACAACCTAC |  |  |
| Aalbdsx♀qR | CATCTACCGATTCTACAG |  |  |
| Aalfru♂qF | GGCATTGAACGGCTACCCT | 55℃，40 | Expression profile analysis for *fru^M^* / *fru^F^* with qPCR |
| Aalfru♂qR | CGTCAGGTTGGACTGATGGT |  |  |
| Aalfru♀qF | CGTGTCCTGTACATTTACCATGC |  |  |
| Aalfru♀qR | TCTCCTCTGTTAGACGGCTG |  |  |
