# Supplementary material for: The AalNix3&4 isoform is required and sufficient to convert Aedes albopictus females into males
Source: PLoS Genet. 2022 Jun 23;18(6):e1010280. doi: 10.1371/journal.pgen.1010280 (PMC9258803; doi:10.1371/journal.pgen.1010280)
Supplement: S2 Table — (DOCX) [file pgen.1010280.s007.docx]

| **S2 Table. Progeny screening of the *AalNix1*-♂2 transgenic line.** | | | | |
| --- | --- | --- | --- | --- |
| **Generation** | **Transgenic^1^** | | **Non-transgenic^1^** | |
|  | **m/m; Nix/+; ♀** | **M/m; Nix/+; ♂** | **m/m; +/+; ♀** | **M/m; +/+; ♂** |
| G_2_ | 8 | 13 | 11 | 10 |
| G_3_ | 21 | 18 | 19 | 22 |
| G_4_ | 112 | 97 | 127 | 119 |
| G_6_ | 40 | 45 | 55 | 42 |
| G_7_ | 152 | 66 | 108 | 83 |
| G_8_ | 212 | 192 | 183 | 172 |
| G_9_ | 150 | 149 | 117 | 94 |
| G_10_ | 374 | 297 | 305 | 258 |
| Total | 1069 | 877 | 925 | 800 |
| 1.1^st^ chromosome genotype: m/m, female, M/m, male; transgene content: Nix/+, hemizygous (one copy), +/+, no copy; morphological phenotype: male, ♂, female, ♀. | | | | |
