# Supplementary material for: The AalNix3&4 isoform is required and sufficient to convert Aedes albopictus females into males
Source: PLoS Genet. 2022 Jun 23;18(6):e1010280. doi: 10.1371/journal.pgen.1010280 (PMC9258803; doi:10.1371/journal.pgen.1010280)
Supplement: S3 Table — (DOCX) [file pgen.1010280.s008.docx]

| **S3 Table. Progeny screening of the *AalNix2*-♂3 transgenic line.** | | | | |
| --- | --- | --- | --- | --- |
| **Generation** | **Transgenic^1^** | | **Non-transgenic^1^** | |
|  | **m/m; Nix/+; ♀** | **M/m; Nix/+;♂** | **m/m; +/+; ♀** | **M/m; +/+; ♂** |
| G_2_ | 12 | 14 | 9 | 11 |
| G_3_ | 35 | 29 | 30 | 28 |
| G_4_ | 128 | 77 | 136 | 129 |
| G_6_ | 132 | 112 | 102 | 97 |
| G_7_ | 109 | 178 | 157 | 150 |
| G_8_ | 224 | 254 | 192 | 185 |
| G_9_ | 93 | 88 | 70 | 83 |
| G_10_ | 411 | 378 | 338 | 307 |
| Total | 1144 | 1130 | 1034 | 990 |
| 1.1^st^ chromosome genotype: m/m, female, M/m, male; transgene content: Nix/+, hemizygous (one copy), +/+, no copy; morphological phenotype: male, ♂, female, ♀. | | | | |
