# Supplementary material for: The AalNix3&4 isoform is required and sufficient to convert Aedes albopictus females into males
Source: PLoS Genet. 2022 Jun 23;18(6):e1010280. doi: 10.1371/journal.pgen.1010280 (PMC9258803; doi:10.1371/journal.pgen.1010280)
Supplement: S4 Table — (DOCX) [file pgen.1010280.s009.docx]

| **S4 Table. Progeny screening of *AalNix3&4*-♂4 transgenic lines.** | | | | | | |
| --- | --- | --- | --- | --- | --- | --- |
| **Generation** | **Transgenic^1^** | | | | **Non-transgenic^1^** | |
|  | **m/m; Nix/+; ♀** | **m/m; Nix/+; intersex** | **m/m; Nix/+; pseudo-male** | **M/m; Nix/+; ♂** | **m/m; +/+; ♀** | **M/m; +/+; ♂** |
| G3 | 0 | 0 | 384 | 14 | 145 | 0 |
| G4 | 0 | 2 | 253 | 7 | 91 | 8 |
| G5 | 0 | 176 | 179 | 90 | 233 | 82 |
| G7 | 0 | 35 | 1 | 44 | 30 | 22 |
| G8 | 0 | 195 | 4 | 119 | 164 | 105 |
| G9 | 0 | 234 | 2 | 251 | 265 | 287 |
| G10 | 0 | 141 | 0 | 173 | 137 | 130 |
| Total | 0 | 783 | 823 | 698 | 1065 | 634 |
| 1.1^st^ chromosome genotype: m/m, female, M/m, male; transgene content: Nix/+, hemizygous (one copy), +/+, no copy; morphological phenotype: male, ♂, female, ♀, intersex. | | | | | | |
