# Supplementary material for: The AalNix3&4 isoform is required and sufficient to convert Aedes albopictus females into males
Source: PLoS Genet. 2022 Jun 23;18(6):e1010280. doi: 10.1371/journal.pgen.1010280 (PMC9258803; doi:10.1371/journal.pgen.1010280)
Supplement: S5 Table — (DOCX) [file pgen.1010280.s010.docx]

| **S5 Table. Average length (millimeters) of the left and right wings of individuals of the WT female, *AalNix3&4*- ♂4 pseudo-male and WT male.** | | | |
| --- | --- | --- | --- |
| **No.** | **WT female** | ***AalNix3&4*-♂4-pseudo-male** | **WT male** |
| 1 | 3.254 | 2.797 | 2.784 |
| 2 | 3.240 | 2.720 | 2.744 |
| 3 | 3.231 | 2.674 | 2.684 |
| 4 | 3.224 | 2.659 | 2.621 |
| 5 | 3.197 | 2.656 | 2.612 |
| 6 | 3.195 | 2.644 | 2.601 |
| 7 | 3.181 | 2.637 | 2.591 |
| 8 | 3.170 | 2.623 | 2.579 |
| 9 | 3.159 | 2.619 | 2.576 |
| 10 | 3.159 | 2.616 | 2.571 |
| 11 | 3.150 | 2.615 | 2.568 |
| 12 | 3.147 | 2.615 | 2.565 |
| 13 | 3.141 | 2.612 | 2.557 |
| 14 | 3.137 | 2.609 | 2.553 |
| 15 | 3.125 | 2.587 | 2.549 |
| 16 | 3.114 | 2.586 | 2.548 |
| 17 | 3.113 | 2.583 | 2.546 |
| 18 | 3.113 | 2.576 | 2.542 |
| 19 | 3.109 | 2.572 | 2.519 |
| 20 | 3.088 | 2.562 | 2.519 |
| 21 | 3.070 | 2.561 | 2.511 |
| 22 | 3.069 | 2.553 | 2.497 |
| 23 | 3.067 | 2.550 | 2.496 |
| 24 | 3.062 | 2.528 | 2.486 |
| 25 | 3.042 | 2.526 | 2.478 |
| 26 | 3.039 | 2.523 | 2.476 |
| 27 | 3.017 | 2.510 | 2.456 |
| 28 | 3.003 | 2.486 | 2.441 |
| 29 | 2.952 | 2.482 | 2.411 |
| 30 | 2.949 | 2.370 | 2.400 |
| Average length(mm) | 3.117 | 2.588 | 2.549 |
