# Supplementary material for: The AalNix3&4 isoform is required and sufficient to convert Aedes albopictus females into males
Source: PLoS Genet. 2022 Jun 23;18(6):e1010280. doi: 10.1371/journal.pgen.1010280 (PMC9258803; doi:10.1371/journal.pgen.1010280)
Supplement: S6 Table — (DOCX) [file pgen.1010280.s011.docx]

| **S6 Table. Progeny screening of *AalNix3&4*-♂4 converted transgenic line.** | | | | |
| --- | --- | --- | --- | --- |
| **Generation** | **Transgenic^1^** | | **Non-transgenic^1^** | |
|  | **m/m; Nix/+; ♂** | **M/m; Nix/+; ♂** | **m/m; +/+; ♀** | **M/m; +/+; ♂** |
| G_3_ | 159 | 0 | 150 | 0 |
| G_4_ | 160 | 0 | 145 | 0 |
| G_5_ | 160 | 0 | 121 | 0 |
| G_7_ | 145 | 0 | 134 | 0 |
| G_8_ | 165 | 0 | 135 | 0 |
| G_9_ | 160 | 0 | 140 | 0 |
| G_10_ | 176 | 0 | 152 | 0 |
| Total | 1125 | 0 | 977 | 0 |
| 1.1^st^ chromosome genotype: m/m, female, M/m, male; transgene content: Nix/+, hemizygous (one copy), +/+, no copy; morphological phenotype: male, ♂, female, ♀. | | | | |
