# Supplementary material for: The AalNix3&4 isoform is required and sufficient to convert Aedes albopictus females into males
Source: PLoS Genet. 2022 Jun 23;18(6):e1010280. doi: 10.1371/journal.pgen.1010280 (PMC9258803; doi:10.1371/journal.pgen.1010280)
Supplement: S7 Table — (DOCX) [file pgen.1010280.s012.docx]

| **S7 Table. Progeny screening of *AalNix3&4*-♂15 transgenic line.** | | | | | | |
| --- | --- | --- | --- | --- | --- | --- |
| **Generation** | **Transgenic^1^** | | | | **Non-transgenic^1^** | |
|  | **m/m; Nix/+; ♀** | **m/m; Nix/+; intersex** | **m/m; Nix/+; convert** | **M/m; Nix/+; ♂** | **m/m; +/+; ♀** | **M/m; +/+; ♂** |
| G3 | 6 | 5 | 1 | 10 | 17 | 21 |
| G4 | 47 | 172 | 11 | 207 | 214 | 232 |
| G6 | 50 | 83 | 5 | 101 | 121 | 112 |
| G7 | 41 | 83 | 2 | 163 | 71 | 85 |
| G8 | 41 | 131 | 0 | 94 | 209 | 233 |
| G9 | 53 | 68 | 0 | 285 | 102 | 105 |
| G10 | 128 | 179 |  |  | 205 | 234 |
| Total | 366 | 721 | 21 | 1033 | 939 | 1022 |
| 1.1^st^ chromosome genotype: m/m, female, M/m, male; transgene content: Nix/+, hemizygous (one copy), +/+, no copy; morphological phenotype: male, ♂, female, ♀, intersex, convert. | | | | | | |
