# Supplementary material for: The AalNix3&4 isoform is required and sufficient to convert Aedes albopictus females into males
Source: PLoS Genet. 2022 Jun 23;18(6):e1010280. doi: 10.1371/journal.pgen.1010280 (PMC9258803; doi:10.1371/journal.pgen.1010280)
Supplement: S8 Table — (DOCX) [file pgen.1010280.s013.docx]

| **S8 Table. DsRed fluorescent phenotypes in progeny resulting from competition assays of *AalNix3&4*-♂4 pseudo-males (m/m; Nix/+) and wild-type males (M/m).** | | | | | | |
| --- | --- | --- | --- | --- | --- | --- |
| **Replicate** | **DsRed+** | **DsRed-** |  | **Replicate** | **DsRed+** | **DsRed -** |
| 1-1 | 0 | 35 |  | 4-1 | 0 | 47 |
| 1-2 | 25 | 30 |  | 4-2 | 35 | 30 |
| 1-3 | 0 | 41 |  | 4-3 | 0 | 50 |
| 1-4 | 0 | 56 |  | 4-4 | 0 | 77 |
| 1-5 | 0 | 63 |  | 4-5 | 0 | 66 |
| 1-6 | 0 | 36 |  | 4-6 | 0 | 9 |
| 1-7 | 0 | 46 |  | 4-7 | 0 | 82 |
| 1-8 | 0 | 35 |  | 4-8 | 42 | 46 |
| 1-9 | 0 | 41 |  | 4-9 | 0 | 74 |
| 1-10 | 0 | 53 |  | 4-10 | 0 | 62 |
| 2-1 | 0 | 41 |  | 5-1 | 37 | 27 |
| 2-2 | 0 | 4 |  | 5-2 | 0 | 59 |
| 2-3 | 0 | 39 |  | 5-3 | 0 | 24 |
| 2-4 | 0 | 41 |  | 5-4 | 0 | 39 |
| 2-5 | 0 | 48 |  | 5-5 | 0 | 62 |
| 2-6 | 0 | 41 |  | 5-6 | 0 | 46 |
| 2-7 | 34 | 33 |  | 5-7 | 22 | 33 |
| 2-8 | 0 | 45 |  | 5-8 | 0 | 44 |
| 2-9 | 0 | 41 |  | 5-9 | 0 | 35 |
| 2-10 | 0 | 65 |  | 5-10 | 0 | 55 |
| 3-1 | 15 | 45 |  | 6-1 | 0 | 53 |
| 3-2 | 0 | 47 |  | 6-2 | 25 | 19 |
| 3-3 | 0 | 42 |  | 6-3 | 0 | 59 |
| 3-4 | 16 | 27 |  | 6-4 | 0 | 54 |
| 3-5 | 0 | 56 |  | 6-5 | 0 | 13 |
| 3-6 | 0 | 37 |  | 6-6 | 0 | 13 |
| 3-7 | 0 | 54 |  | 6-7 | 0 | 28 |
| 3-8 | 0 | 32 |  | 6-8 | 0 | 13 |
| 3-9 | 0 | 54 |  | 6-9 | 0 | 15 |
| 3-10 | 0 | 9 |  | 6-10 | 0 | 41 |
