# Supplementary material for: The AalNix3&4 isoform is required and sufficient to convert Aedes albopictus females into males
Source: PLoS Genet. 2022 Jun 23;18(6):e1010280. doi: 10.1371/journal.pgen.1010280 (PMC9258803; doi:10.1371/journal.pgen.1010280)
Supplement: S9 Table — (DOCX) [file pgen.1010280.s014.docx]

| **S9 Table. DsRed fluorescent phenotypes in progeny resulting from competition assays of wild-type males (M/m) with the *AalNix3&4*-♂4 transgene and wild-type (M/m) males.** | | | | | | |
| --- | --- | --- | --- | --- | --- | --- |
| **Replicate** | **DsRed+** | **DsRed-** |  | **Replicate** | **DsRed+** | **DsRed-** |
| 1-1 | 7 | 21 |  | 4-1 | 24 | 16 |
| 1-2 | 0 | 42 |  | 4-2 | 9 | 35 |
| 1-3 | 23 | 26 |  | 4-3 | 0 | 54 |
| 1-4 | 14 | 26 |  | 4-4 | 9 | 8 |
| 1-5 | 16 | 17 |  | 4-5 | 14 | 17 |
| 1-6 | 0 | 30 |  | 4-6 | 0 | 32 |
| 1-7 | 0 | 64 |  | 4-7 | 14 | 12 |
| 1-8 | 26 | 21 |  | 4-8 | 10 | 25 |
| 1-9 | 0 | 0 |  | 4-9 | 13 | 26 |
| 1-10 | 0 | 0 |  | 4-10 | 0 | 0 |
| 2-1 | 0 | 65 |  | 5-1 | 22 | 25 |
| 2-2 | 0 | 75 |  | 5-2 | 0 | 17 |
| 2-3 | 0 | 0 |  | 5-3 | 12 | 16 |
| 2-4 | 0 | 0 |  | 5-4 | 22 | 20 |
| 2-5 | 36 | 33 |  | 5-5 | 20 | 13 |
| 2-6 | 19 | 17 |  | 5-6 | 0 | 0 |
| 2-7 | 27 | 28 |  | 5-7 | 13 | 30 |
| 2-8 | 0 | 31 |  | 5-8 | 0 | 63 |
| 2-9 | 12 | 22 |  | 5-9 | 0 | 3 |
| 2-10 | 0 | 0 |  | 5-10 | 6 | 13 |
| 3-1 | 15 | 12 |  | 6-1 | 17 | 29 |
| 3-2 | 29 | 39 |  | 6-2 | 15 | 13 |
| 3-3 | 23 | 20 |  | 6-3 | 0 | 18 |
| 3-4 | 7 | 18 |  | 6-4 | 0 | 6 |
| 3-5 | 6 | 10 |  | 6-5 | 21 | 20 |
| 3-6 | 24 | 19 |  | 6-6 | 24 | 24 |
| 3-7 | 19 | 23 |  | 6-7 | 14 | 18 |
| 3-8 | 21 | 19 |  | 6-8 | 0 | 37 |
| 3-9 | 0 | 20 |  | 6-9 | 0 | 0 |
| 3-10 | 0 | 0 |  | 6-10 | 0 | 0 |
